# Longitudinal *in vivo* MRI in a Huntington’s disease mouse model: Global atrophy in the absence of white matter microstructural damage

**DOI:** 10.1038/srep32423

**Published:** 2016-09-01

**Authors:** Jessica J. Steventon, Rebecca C. Trueman, Da Ma, Emma Yhnell, Zubeyde Bayram-Weston, Marc Modat, Jorge Cardoso, Sebastian Ourselin, Mark Lythgoe, Andrew Stewart, Anne E. Rosser, Derek K. Jones

**Affiliations:** 1 Cardiff University Brain Research Imaging Centre, School of Psychology, Cardiff University, Park Place, Cardiff, CF10 3AT, UK; 2 Brain Repair Group, Life Science Building, 3rd Floor, School of Biosciences, Cardiff University, Museum Avenue, Cardiff, CF10 3AX, UK; 3 Neuroscience and Mental Health Research Institute, Cardiff University, Hadyn Ellis Building, Cathays, Cardiff, CF24 4HQ, UK; 4 Experimental MRI Centre, School of Biosciences, Cardiff University, Museum Avenue, Cardiff, CF10 3AX, UK; 5 School of Life Sciences, Queen’s Medical Centre, Nottingham University, Nottingham, NG7 2UH, UK; 6 Centre for Medical Imaging Computing, University College London, London, UK; 7 Centre for Advanced Biomedical Imaging, Division of Medicine, University College London, London, UK; 8 Institute of Psychological Medicine and Neurology, School of Medicine, Hadyn Ellis Building, Maindy Road, Cathays, Cardiff CF24 4HQ, UK

## Abstract

Huntington’s disease (HD) is a genetically-determined neurodegenerative disease. Characterising neuropathology in mouse models of HD is commonly restricted to cross-sectional *ex vivo* analyses, beset by tissue fixation issues. *In vivo* longitudinal magnetic resonance imaging (MRI) allows for disease progression to be probed non-invasively. In the HdhQ150 mouse model of HD, *in vivo* MRI was employed at two time points, before and after the onset of motor signs, to assess brain macrostructure and white matter microstructure. *Ex vivo* MRI, immunohistochemistry, transmission electron microscopy and behavioural testing were also conducted. Global brain atrophy was found in HdhQ150 mice at both time points, with no neuropathological progression across time and a selective sparing of the cerebellum. In contrast, no white matter abnormalities were detected from the MRI images or electron microscopy images alike. The relationship between motor function and MR-based structural measurements was different for the HdhQ150 and wild-type mice, although there was no relationship between motor deficits and histopathology. Widespread neuropathology prior to symptom onset is consistent with patient studies, whereas the absence of white matter abnormalities conflicts with patient data. The myriad reasons for this inconsistency require further attention to improve the translatability from mouse models of disease.

Huntington’s disease (HD) is an autosomal dominant neurodegenerative disease caused by a polyglutamine (CAG) repeat expansion on the huntingtin (HTT) gene^1^. The discovery of the mutant HTT gene led to the development of genetic animal models which have a similar genetic defect to that seen in the human disease, allowing for the study of early pathological, cellular and molecular alterations caused by the mutation. Animal models provide a direct measure of neuropathology not possible in patients *in vivo*, and in comparison to patient cohorts, animal models can be experimentally controlled in terms of disease stage, number of CAG repeats and genetic background, reducing the inter-subject variance.

The longitudinal characterisation of HD mouse lines is crucial to understanding the timing, nature and severity of phenotype progression^2^ and ultimately to be able to translate this to the human disease. A major benefit of animal models is that the whole disease course can be studied; in patients it is difficult to study structural brain changes *in vivo* in more advanced disease stages due to the incompatibility of chorea with magnetic resonance imaging (MRI). The majority of MRI studies using mouse models of HD have been cross-sectional. However, the primary value of pre-clinical (animal) MRI lies in the capability for within-subject longitudinal designs with increased power and experimental control to study the disease time course and evaluate therapeutic outcomes at different disease stages. Longitudinal MRI enables a region- and temporally-specific readout of neuropathology, which can inform on the design of future animal studies, allowing for the most appropriate mouse line to be selected.

Although the hallmark of HD is a selective loss of medium spiny neurons in the striatum, brain imaging studies have revealed white matter abnormalities at all disease stages in patients with HD^3^,^4^,^5^,^6^. The use of animal models allows the cellular basis of these abnormalities to be explored, and recent work in the HdhQ140 model revealed increased axonal swelling at 12 months but not 6 months of age in homozygote Hdh^Q140/Q140^ mice, with no axonal pathology in heterozygote mice^7^. In the transgenic YFP(J16)-R6/2 model, marked degeneration of callosal axons was found prior to the onset of motor signs^8^. In contrast, previous work in a mouse model with a different YFP expression pattern (YFP(H)-R6/2) found no axonal disruption^7^, suggesting that axonal pathology is spatially and temporally selective and dependent on the specific genetic model of HD.

Diffusion MRI is a powerful method for non-invasive characterization of tissue microstructure, and is a valuable addition to longitudinal designs. However, whereas diffusion MRI is routinely used in human studies, it is technically challenging in the mouse brain, which is approximately 1000 times smaller. One issue is multiple white matter fibre orientations within a single imaging voxel, which cannot be resolved using the conventional diffusion tensor model^9^. We have shown that crossing fibres within an imaging voxel are present in the mouse brain (see Supplementary Figure S1), thus, using more sophisticated approaches which are capable of resolving crossing fibres will improve the sensitivity of diffusion tractography in the mouse brain.

Previous studies have used MRI to characterise neuropathological changes in transgenic mouse models of HD^10^,^11^ whereas in this work, we utilize a knock-in model where the genetic basis of HD is more precisely recapitulated. The HdhQ150 mouse model^12^ was created by directly replacing the normal mouse endogenous CAG repeat length with an expanded 150 CAG sequence in the mouse Htt gene without affecting the rest of the endogenous sequence of the gene. The gene is not over-expressed and the use of the endogenous Hdh promoter means that a similar expression profile to that seen in the human condition is produced. The behavioural deficits of *Hdh*^Q150/Q150^ mice have previously been well characterised longitudinally^2^,^13^ with cognitive deficits evident prior to motor abnormalities just as in the human disease. *Ex vivo* histological analysis has shown that *Hdh*^*Q150/Q150*^ mice display many of the characteristics of the human disease including Htt aggregation that is most marked in the striatum but also evident to a lesser degree in the cortex, thalamus and hippocampus, along with neuronal intra-nuclear inclusions^12^,^14^. These pathological changes are evident from around 5 months of age followed by striatal cell loss at 8 months of age^14^, prior to the onset of motor signs, in line with the human literature from the pre-symptomatic disease stage.

Despite firm evidence for grey matter pathology, it remains unclear whether white matter degeneration occurs in the HdhQ150 mouse model of HD. To address this question, we used *in vivo* MRI as a tool to investigate both macrostructure and microstructure pathology at both an early and late time point in the disease course.

## Results

### Reduced body weight in Hdh^Q150/Q150^ mice

**Figure 1:**
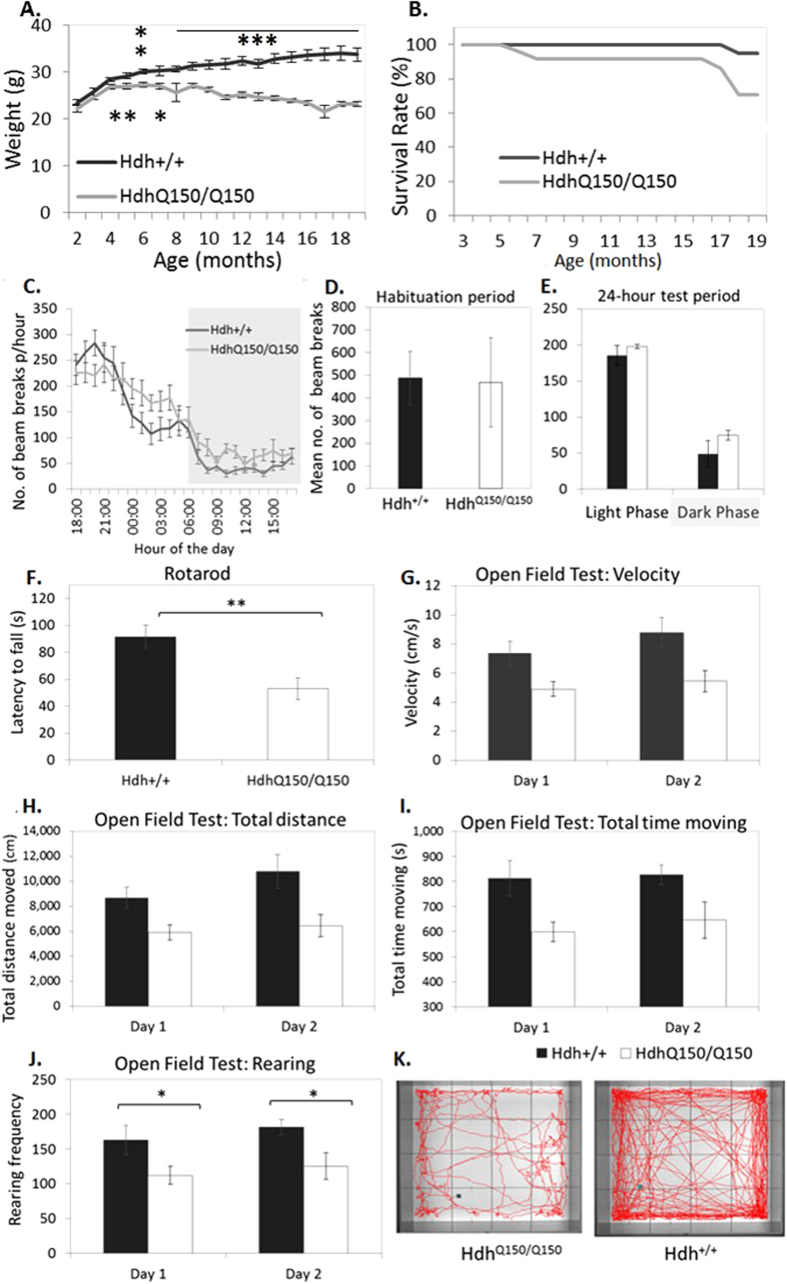
Behavioural Assessment at 19 months old. (**A**) Body weight. (**B**) Survival rates excluding cases related to anaesthesia. (**C**) Total number of beam breaks/hour over 24-hours. (**D**) Average number of beam breaks in the first 2 hours, and E: during the light and dark phase. (**F**) Latency to fall on Rotarod. (**G–J**) Open Field Test on consecutive days. (**F**) Movement maps made over 20-minutes by a single Hdh^Q150/Q150^ mouse and single Hdh^+/+^ mouse. Error bars = ± standard error of the mean. *p < 0.05, **p < 0.01, ***p < 0.001.

A genotype × time interaction was found for body weight (*Greenhouse-Geisser corrected* F_12.04,145.89_ = 27.30, p < 0.001, ε = 0.179, Fig. 1). *Hdh*^*Q150/Q150*^ mice had reduced body weight compared to *Hdh*^+/+^ mice from 3 months of age (all p < 0.05). Modest non-significant correlations (r > 0.3, p > 0.05) with weight were found for the Open Field measures and for locomotion over 24 hours.

### Survival and recovery from anaesthesia

There was no difference in survival between *Hdh*^+/+^ and *Hdh*^*Q150/Q150*^ from 0–7 months (χ (1, n = 50) = 1.02, p > 0.05) and from 7–19 months (χ (1, n = 44) = 1.0, p > 0.05). Genotype did not affect recovery from anaesthesia during the MRI session at 7 months old (χ (1, n = 49) = 0.27, p > 0.05), however at 19-months, *Hdh*^*Q150/Q150*^ mice were less likely to survive the scan (χ (1, n = 38) = 4.02, p < 0.05). Late stage behavioural tests and histological analyses are therefore based on correspondingly reduced and unequal group sizes.

### Motor deficits are evident at 19 months old

Descriptive statistics are shown in Fig. 1.

#### Circadian-related activity

Habituation activity was measured over the first 2 hours; circadian-related activity was assessed over 24 hours. There was no effect of genotype on activity levels (F_1,26_ = 0.003, p > 0.05). With weight as a covariate, *Hdh*^*Q150/Q150*^ mice were more active than *Hdh*^+/+^ mice during both the light and dark phase (F_1,26_ = 5.26 and 4.49 respectively, p < 0.05).

#### Rotarod

Averaged over 2 trials, *Hdh*^*Q150/Q150*^ mice had a significantly shorter latency to fall from the rod, t_27_ = 3.08, p < 0.01.

#### Open Field Test

Total distance moved, time spent moving, velocity, and rearing frequency (standing on rear limbs) were significantly reduced in *Hdh*^*Q150/Q150*^ mice (t_20_ = 2.39, 2.42, 2.31 and 2.75 respectively, p < 0.05). With body weight as a covariate, the group differences were not significant, all p > 0.05. Analysis of the univariate main effects revealed that the difference was driven by *Hdh*^*Q150/Q150*^ mice engaging in rearing behaviour less frequently than *Hdh*^+/+^ mice (F_1,22_ = 10.08, p < 0.05).

### Global atrophy in Hdh^Q150/Q150^ mice

Longitudinal *in vivo* MRI was conducted to measure volume loss and change in the HdhQ150 mouse brain. Atlas-based segmentation was used to delineate regions of interest (ROIs). The total brain volume (TBV) of *Hdh*^*Q150/Q150*^ mice was significantly reduced compared to *Hdh*^+/+^ mice at both 7 months (−5.66%) and 19 months of age (−11.68%). After normalising the regional volumes for TBV, there were no volumetric differences between hemispheres (all p > 0.05), so the volumes of the left and right ROIs were summed.

**Figure 2:**
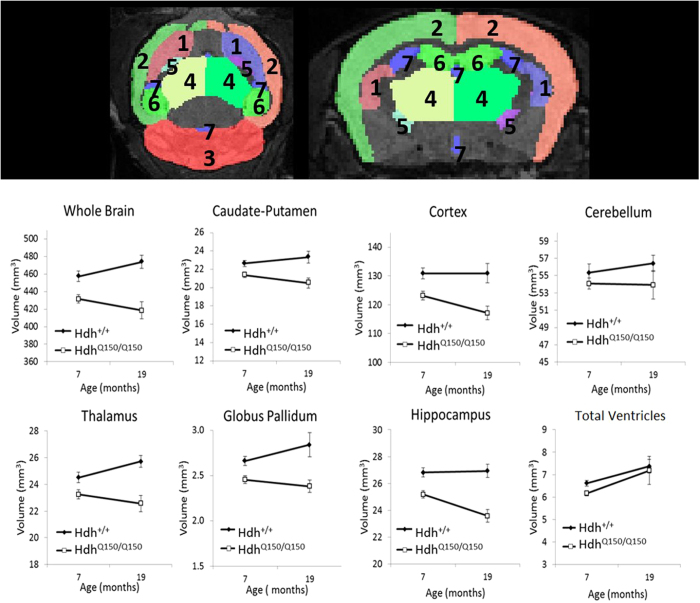
Longitudinal *in vivo* MRI atlas-based segmentation of T_2_-weighted images. Top panel shows segmented ROI masks in the axial and coronal plane ([1] caudate-putamen; [2] cortex; [3] cerebellum; [4] thalamus; [5] globus pallidum; [6] hippocampus; [7] ventricles) overlaid on a T_2_-weighted image from a 20-month old wild-type mouse. Absolute volumes presented as means ± SEM.

The absolute volume of the caudate-putamen, thalamus, hippocampus, globus pallidum and cortex was significantly reduced in *Hdh*^*Q150/Q150*^ mice at both ages (all p < 0.05, see Fig. 2 and Table 1). However, there was no difference between mice in regional volumes normalised by TBV to account for global atrophy (p > 0.05, Table 1), with the exception of the cerebellum, which was selectively spared at both ages in *Hdh*^*Q150/Q150*^ mice. This suggests that volume loss in the HdhQ150 mouse brain is global rather than region-specific and neuropathology occurs prior to the onset of motor signs.

**Figure 3:**
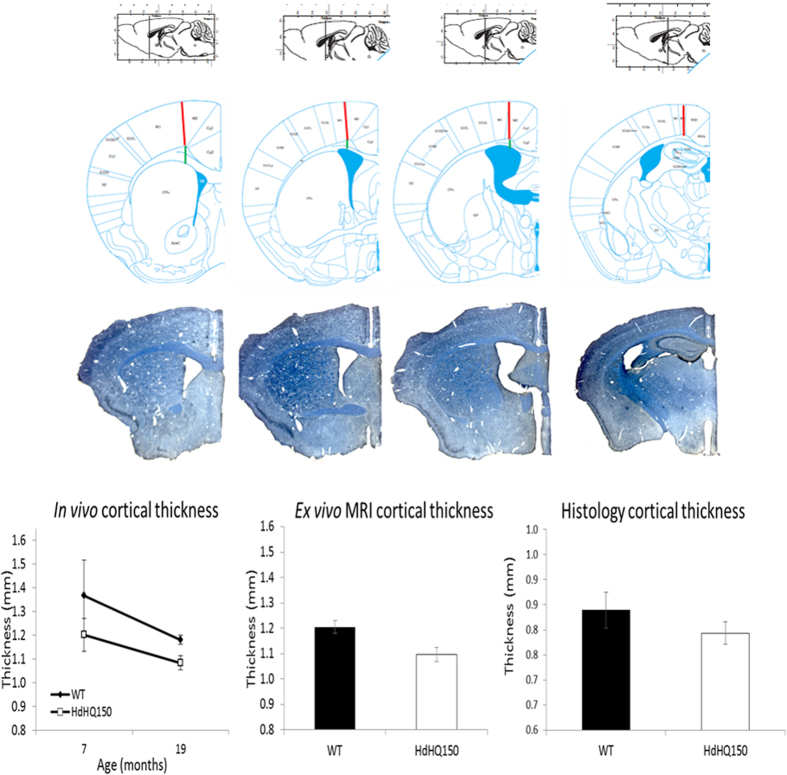
Top. Approximate Bregma positions for cortical thickness measures (red line = measurement). Bottom sections show representative stained sections for cortical thickness measures (blue = myelin). Graphs show cortical thickness measures from MRI images at different ages (left) and myelin-stained 40 μm brain sections at 20 months old (right). WT: Hdh^+/+^ mice; HdhQ150: HdhQ150/Q150 mice.

In contrast to cortical volume loss, there was no difference in absolute or normalised cortical thickness in *Hdh*^*Q150/Q150*^ mice at 7 months old (F_1,42_ = 1.37, p > 0.05, Fig. 3). At 19 months old, the motor cortices in the *Hdh*^*Q150/Q150*^ mice were 8.7% thinner (F_1,26_ = 12.49, p < 0.01), whereas there was no effect of genotype on cortical thickness normalised by TBV (F_1,26_ = 0.47, p > 0.05).

### Global volume loss is not progressive beyond 7 months in Hdh ^Q150/Q150^ mice

Despite the smaller sample size at 19-months old (*Hdh*^*Q150/Q150*^ n = 10, *Hdh*^+/+^ n = 17), a repeated-measures ANOVA examined the effect of time on volume. TBV did not change with time (F_1,25_ = 0.43, p > 0.05) although there was a trend towards a time × genotype interaction (F_1,25_ = 7.75, p = 0.01 uncorrected, p > 0.05). Ventricular volume increased with time (F_1,25_ = 7.70, p < 0.05). There was no effect of time on absolute volume of the caudate-putamen (F_1,25_ = 2.65, p > 0.05), thalamus (F_1,24_ = 0.04, p > 0.05), hippocampus (F_1,25_ = 2.26, p > 0.05), globus pallidum (F_1,22_ = 0.08, p > 0.05), and cortex (F_1,25_ = 2.38, p > 0.05). For all ROIs, with the exception of the cerebellum and ventricles, regional atrophy was found in Hdh^Q150/Q150^ mice (all p < 0.005).

**Figure 4:**
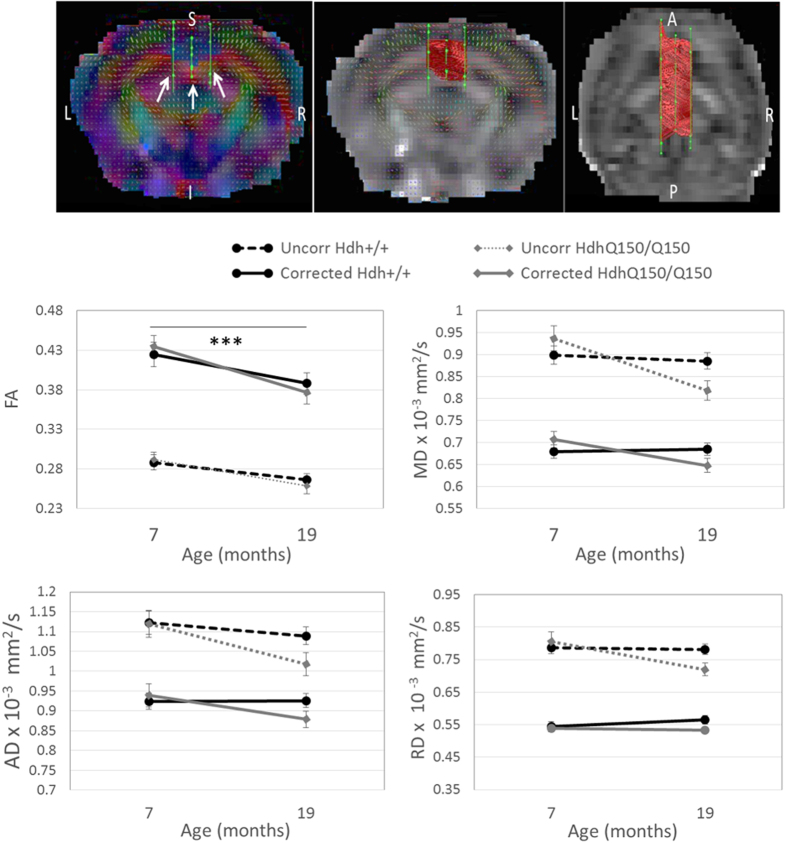
Tractography of the corpus callosum. Top Left: arrows indicate ROI placement in the coronal plane overlaid on a map colour-coded for the principal diffusion vector. Top middle (coronal) and top right (axial) panes shows tracts overlaid on a single subject FA map. Line graphs show DTI metrics before and after partial volume correction. Uncorr = uncorrected data, ***p < 0.005. N = 22/22 at 7-months; N = 19/14 at 19-months, for Hdh^+/+^/Hdh^Q150/Q150^ respectively.

From the MRI images, cortical thinning was found from 7- to 19-months old (F_1,24_ = 7.09, p < 0.05, Fig. 4), with a main effect of genotype (F_1,24_ = 13.30, p < 0.05) and no interaction between genotype and time, suggesting age affected both groups equally. Time interacted with cortical region; the most anterior cortical region (Bregma 1.10mm) was significantly thinner over time (F_1,24_ = 21.58, p < 0.001), with a more modest effect of time at Bregma −0.22 mm (F_1,24_ = 11.48, p < 0.01) and no difference at Bregma −1.06 mm and 0.14 mm (both p > 0.05).

### *Ex vivo* evidence

For higher-resolution *ex vivo* images acquired at 20 months old, the cerebellum and brain stem were not included in the TBV measure due to artefacts related to *ex vivo* preparation, thus TBV is not directly comparable to the *in vivo* measurements. There were no genotype differences in TBV in the *ex vivo* images (4.08%, F_1,34_ = 1.70, p > 0.05). As with the *in vivo* MRI results, after accounting for TBV there were no laterality effects (all p > 0.05), so the left and right ROIs were summed. There were no genotype differences in the absolute or normalised volume of the caudate-putamen, thalamus, globus pallidum, neocortex, hippocampus and the ventricles (all p > 0.05).

In accordance with the *in vivo* results, the absolute motor cortices measured from *ex vivo* MRI images were 9.3% thinner in *Hdh*^*Q150/Q150*^ mice (F_1,33_ = 12.57, p < 0.005, Fig. 4). The motor cortices in the histologically stained sections were 5.4% thinner in *Hdh*^*Q150/Q150*^ mice compared to *Hdh*^+/+^ mice, but this did not meet statistical significance (F_1,13_ = 0.48, p > 0.05).

### Tissue fixation produces lower estimates

Absolute volume was smaller in the *ex vivo* images at 20-months compared to the i*n vivo* images acquired at 19-months in the caudate-putamen (−6.8%, t_24_ = −2.19, p < 0.05), thalamus (−7.3%, t_23_ = −4.21, p < 0.001) and hippocampus (−9.02%, t_24_ = −3.68, p < 0.01). There was no difference in the cortex (0.3%, t_19_ = −1.0, p > 0.05), and an increase in globus pallidum volume (15%, t_24_ = 3.17, p < 0.01). The cerebral cortices were thinner when measured in stained tissue sections compared to *ex vivo* MRI (Wilks’ λ = 0.117, F_1,12_ = 90.37, p < 0.001).

### HdhQ150 mice do not exhibit white matter microstructural abnormalities

Because of the sample size difference, white matter microstructure in the corpus callosum was examined separately at each age using t-tests. Mean values are shown in Fig. 4; at 7-months of age, prior to the onset of motor signs, there was no difference in tensor-based metrics, all p > 0.05. At 19-months of age when motor signs were evident, Hdh^Q150/Q150^ mice had reduced mean diffusivity (MD; t_31_ = 2.31, p = 0.028 uncorrected) and radial diffusivity (RD, t _31_ = 2.43, p = 0.021 uncorrected) compared to Hdh^+/+^ mice, however these differences did not survive multiple comparison correction (FDR-adjusted p-value > 0.05). Importantly, when the same statistical analyses were performed on tensor metrics corrected for CSF-related contamination^15^, the diffusivity differences observed between HDh^Q150/Q150^ and Hdh^+/+^ were no longer significant (see Fig. 4 for comparison). For all DTI metrics and at both time points, there was a significant difference between uncorrected and corrected DTI metrics (all p < 0.001). This demonstrates the importance of accounting for partial volume effects when studying white matter microstructure in the presence of atrophy.

The free water fraction did not differ between *Hdh*^*Q150/Q150*^ and *Hdh*^+/+^ mice at 7 months (0.282  ± 0.011 and 0.281 ± 0.010 respectively, t_41_ = −0.077, p > 0.05), or 19 months (0.239 ± 0.014 and 0.253 ± 0.011 respectively; t_29_ = 0.790, p > 0.05). Albeit with a reduce sample size, a repeated measures ANOVA assessed change over time. Fractional anisotropy (FA) was significantly reduced over time for both corrected and uncorrected data (F_1,27_ = 16.17 and 32.51 respectively, both p < 0.005), with no change in MD. There was a trend for a reduction in axial diffusivity (AD; λ1) at 19-months compared to 7-months in the uncorrected data (F_1,29_ = 7.64, p = 0.01 uncorrected, FDR-adjusted p > 0.05), whereas there was no effect of time in corrected AD values (F_1,27_ = 1.84, p > 0.05). For all DTI measures, there was no interaction between genotype and time (all p > 0.05). Similarly, from the electron microscopy images, axon density in the genu, body and splenium of the corpus callosum was not affected by genotype (Table 2, all p > 0.05). The percentage of decompacted axons was highly variable in both groups of mice, with no genotype differences (all p > 0.05).

### Relationship between neuropathology and behaviour

To reduce the number of planned comparisons, a correlational analysis probed the association between structure and motor measures where deficits were found in the *Hdh*^*Q150/Q150*^ mice. Free water fraction in the corpus callosum was negatively correlated with rearing frequency (normalised for body weight) on the Open Field task in *Hdh*^+/+^ mice (r = −0.574, p < 0.05) and not in *Hdh*^*Q150/Q150*^ mice, p > 0.05.

Similarly, locomotion over 24-hours correlated with cortical volume for *Hdh*^+/+^ mice (r = 0.642, p < 0.05) but not *Hdh*^*Q150/Q150*^ mice, r = 0.304, p > 0.05. Rotarod performance in the *Hdh*^*Q150/Q150*^ mice was associated with normalised thalamus volume (r = −0.834, p < 0.05), however the direction of effect was counterintuitive, with superior rotarod performance associated with reduced thalamus volume.

## Discussion

Neuropathology in the HdhQ150 mouse model of HD has previously been characterised using *ex vivo* techniques^2^ beset by methodological issues associated with tissue fixatives and inherently limited to cross-sectional designs. In this first application of *in vivo* MRI to the HdhQ150 model, total brain volume (TBV) was reduced at 7-months, prior to the onset of motor signs, and at 19-months of age, when motor signs were present. After accounting for global atrophy, localised atrophy and cortical thinning were not evident and the cerebellum was relatively spared at both time points. White matter microstructural abnormalities were not detected in the corpus callosum at any age, in contrast to patient work and *ex vivo* analyses in alternative HD models^7^,^8^.

The MRI analyses provides additional, unique information to histology, examining whole structures rather than selective slices through a structure. The results are in accordance with histological work in the same mouse model^14^, and further show that volume loss is global and not specific to the striatum from 7 months of age. The selective sparing of the cerebellum fits with an anterior-posterior pattern of pathology^2^ although atrophy in specific cerebellar subregions has been observed in symptomatic HD patients^16^. The globalised atrophy observed agrees with work in pre-symptomatic gene carriers, where a smaller intracranial brain volume was found^17^.

*Hdh*^*Q150/Q150*^ mice had prominent motor deficits at 19-months. A novel finding was a relationship between rearing frequency and free water fraction along the corpus callosum, reconstructed from *in vivo* diffusion MRI data, in *Hdh*^+/+^ mice but not *Hdh*^*Q150/Q150*^ mice, suggesting a disturbed relationship between white matter structure and motor function in HD. MRI provides a unique opportunity to assess free water in the intact brain, and previous work has shown altered free water in HD^5^, schizophrenia^18^, brain injury^19^ and Parkinson’s disease^20^. Counterintuitively, larger thalami were associated with a shorter latency to fall on the rotarod specifically in *Hdh*^*Q150/Q150*^ mice. The direction of effect highlights the difficulty in interpreting volume measures which may be sensitive to numerous pathological processes aside from cell loss including cellular swelling or other inflammatory responses. This demonstrates that whereas MRI is highly sensitive to neuropathology, *ex vivo* histological measures provide biological specificity not currently possible with *in vivo* MRI.

No white matter microstructural changes were found in the *Hdh*^*Q150/Q150*^ mice despite corpus callosum abnormalities reported in pre-symptomatic and symptomatic HD participants^5^,^21^. White matter anatomy differs between species^22^ and it is unclear if functional subdivisions exist in the mouse corpus callosum which may dilute effects when analysed as a homogenous structure. The lack of difference in axon density and myelin structure from the microscopy analysis corroborates the MRI result and suggests that the null finding is not due to insufficient sensitivity. However, the statistical tests conducted at the second *in vivo* time point showed a trend towards changes in the diffusivity metrics (MD and RD) in the corpus callosum. MD and RD were both reduced in Hdh^Q150/Q150^ mice although this difference was not significant after adjusting the p-value for multiple comparisons and CSF-related partial volume contamination. This highlights the importance of correcting for partial volume for reliable white matter microstructure characterisation. The results suggest that in comparison to marked grey matter atrophy early in the disease course, white matter abnormalities may be more subtle and occur later, requiring larger sample sizes to detect more modest effects. The microscopy analyses found no difference in axon density, count or myelin decompaction, yet further studies with higher-resolution images are necessary to examine myelin sheath thickness.

The lack of localised atrophy after accounting for global volume contrasts with patient studies^23^. A change in volume over 12-months in the mouse does not readily translate to a period of time in humans, and conceivably an earlier time point in the mouse was necessary to capture localised changes. Interpreting reduced brain volume in terms of biophysical changes and in relation to previous immunohistochemistry findings is not straightforward due to differences in scale. Whereas the number of intra-nuclear inclusions increased with time in *Hdh*^*Q150/Q150*^ mice^14^, neuronal count was reduced from 6 months of age with no continued decline^14^. This absence of a progressive phenotype beyond 6 months combined with diffuse mutant *Htt* staining from an early age supports the pattern of results we observed.

Whole brain atrophy was found to be stable across time which may suggest that either widespread atrophy occurs early and is not progressive, or that the mutant gene affects development. A smaller intracranial volume has been reported in pre-symptomatic HD participants^17^ however progressive whole brain volume loss is evident in pre-symptomatic and symptomatic HD participants^24^. Compared to *Hdh*^+/+^ mice, *Hdh*^*Q150/Q150*^ mice did not reach a normal mature adult weight, which, in combination with global brain atrophy at 7 months, may indicate abnormal developmental processes. The concept of mutant HTT affecting developmental processes is not new^25^ with HTT regulating cortical neurogenesis^26^. Thus, further study should be directed toward exploring the contribution of neurodevelopmental processes in HD.

Despite no difference in lifespan, *Hdh*^*Q150/Q150*^ mice were less tolerant of anaesthesia required for the MRI acquisition at 19-months old, with a significantly higher mortality rate. This could be explained by numerous factors, including a change in oxygen consumption rate affecting survival for hypoxemia, a difference in metabolic rate, thermoregulatory efficiency, or a difference in hepatic glycogen reserves in the aged *Hdh*^*Q150/Q150*^ mice. As mutant *Htt* is expressed throughout the entire body, it is likely that deficits in hepatic, renal and/or cardiac systems affected the response to anaesthesia; cardiac abnormalities have recently been shown in *Hdh*^*Q150/Q150*^ mice^27^, and hepatic dysfunction is an area of recent research attention in HD^28^. This highlights that the suitability of an anaesthetic procedure is dependent on mouse strain, age and weight^29^ which is problematic in longitudinal studies where age and weight are not fixed.

*Ex vivo* volumetric measurements were significantly lower compared to *in vivo* measures, with some regions more severely affected, and were less sensitive to genotype effects. The chemical fixation and staining process resulted in a reduced sample size due to the removal of poor quality samples from the analysis, which may explain the different statistical findings. This suggests that a sole reliance on *ex vivo* techniques may underestimate or distort the spatial and temporal pattern of neuropathology. Whereas the MRI measurements came from both hemispheres, the immunohistochemistry and electron microscopy were conducted on one hemisphere each which can increase tissue damage during processing.

## Concluding Comment

Here we demonstrate that longitudinal *in vivo* MRI has the sensitivity to detect disease-related macrostructural changes in a knock-in mouse model of HD. There was no evidence for white matter microstructural abnormalities at any age, validated with electron microscopy analyses, which contrasts with the patient literature. This may suggest that the precision of genetic models to recapitulate neuropathology varies with tissue type. As with previous work^10^, the link between neuropathology and behavioural deficits was not clear, with evidence for a disturbed structure-function relationship in the HD mice for motor coordination. Investigations into other symptom modalities are required to ascertain the extent of structure-function disturbances.

## Methods

### Ethics statement

All procedures followed protocols in accordance with the United Kingdom Animals (Scientific Procedures) Act of 1986, approved by Cardiff University Ethical Review Process Committee, carried out under Home Office License 30/3036.

### Animals

Homozygous *Hdh*^*Q150/Q150*^ mice were bred in-house using a heterozygous mutant × heterozygous mutant breeding regime on the 129/Ola × C57BL6/J background^12^. Genotype was confirmed by tail tipping (Laragen Inc., Los Angeles, USA). Mean CAG repeat length on allele 1 was 147 ± 7 SD and 158 ± 5 on allele 2. In total, 25 *Hdh*^*Q150/Q150*^ male mice and 25 age-matched Hdh^+/+^ male mice were used.

Mice were housed in mixed genotype groups of between 1 and 3 mice with moderate enrichment, and subject to a 12-hour light: dark cycle with controlled room temperature (21 ± 3 °C) and relative humidity (60 ± 3%). 7-months of age was selected to represent the pre-symptomatic disease stage, with the absence of motor signs; and 19 months of age was selected as the symptomatic time point. Fighting between cagemates due to long separation times during MRI scanning occurred at 7 months, thus all mice were individually housed thereafter. Mice were weighed monthly as progressive weight loss is a sign of disease progression and is a core feature of these mouse models^30^.

### Magnetic Resonance Imaging

The experimental design is shown in Supplementary Figure 3. Data were acquired on a 9.4 Tesla small bore (20 cm) Bruker Biospin system, equipped with BGA12-S (12 cm inner bore size, integrated shims) gradients. A transmit 1H 500 Watt echo-planar imaging (EPI) volume coil was used with a phased array 4-channel surface coil and Paravision software (version 5.1, Bruker Biospin).

### *In vivo* longitudinal MRI

To improve animal health and recovery, 500 μl of glucose saline was administered via sub-cutaneous injection immediately prior to, and immediately after the MRI session. Animals were anaesthetised with isofluorane (5% for induction, 1.8–2.2% for maintenance, mixed with 100% oxygen) delivered at ~1 litre/minute. At 19 months, the long scan time was poorly tolerated, thus the scan was split into two sessions one week apart.

A 3D Turbo Rapid Acquisition with Refocused Echoes (RARE) T_2_-weighted scan was used both *in vivo* and *ex vivo.* The *in vivo* in-plane resolution was 120 × 120 μm with a field of view (FOV) of 1.54 × 1.54 cm, matrix of 128 × 128 × 64, TR/TE = 1750/17.5 ms, bandwidth 50000 Hz, RARE factor of 4, 1 average, and an acquisition time of approximately 1 hour 8 minutes. For the *ex vivo* sequence, the image resolution was 60 μm^3^ with the following parameters: FOV 1.54 cm × 1.54 cm × 1.02 cm, matrix = 256 × 256 × 168, TR/TE = 1750/17.5 ms, bandwidth 50000 Hz, RARE factor of 4, 3 averages, with an acquisition time of 11 hours.

At both *in vivo* time points, a multi-shot DTI echo-planar imaging (EPI) MRI sequence was acquired with 27 contiguous axial slices of 320 μm in thickness. Navigator echoes minimised distortion artefacts and 2 averages increased the SNR. The in-plane resolution was 213 μm × 213 μm at a FOV of 2.24 cm × 2.24 cm and an acquisition matrix of 96 × 96. A partial Fourier acquisition (acceleration factor = 1.35, 23 overscan lines) was used to reduce the acquisition time. 4 dummy scans were used to achieve magnetisation equilibrium. Respiratory-gating was used to reduce motion artefacts. Data were acquired with a b-value of 1000 s/mm^2^ applied over 30 isotropically-distributed diffusion-weighted directions (Jones 30^31^) with 3 b0 images, TR/TE = 8500/18.70 ms, δ/Δ = 4/9 ms. The average acquisition time was 1 hour.

### Deterministic Tractography

Post-processing of diffusion MRI images was conducted using ExploreDTI (version 4.8.3) software^32^ as previously described in Steventon *et al.*^5^. Briefly, images were corrected for distortions due to motion, eddy currents and field inhomogeneity^33^ and any rotations applied were also applied to the encoding vector^34^ and the signal intensity was modulated by the Jacobian determinant of the transformation^35^. Images were corrected for contamination due to atrophy-related free water^15^.

Whole-brain deterministic tracking was conducted using a home-written software plugin to perform damped Richardson-Lucy deconvolution^36^ (DRL), a spherical deconvolution approach capable of resolving crossing fibres. The tracking algorithm estimated the fibre orientation distribution function (fODF) at each seed point and propagated in 0.05 mm steps along this direction, with an angle threshold of 40°. Three-dimensional corpus callosum reconstructions were extracted from the whole-brain tractograms using multiple ROIs drawn manually in native space by a single blinded operator (JJS). An initial ROI was drawn on the mid-sagittal plane; the analysis was restricted to corpus callosal fibres using two additional ‘AND’ gate ROIs (which segment fibres that traverse both ROIs) drawn in the sagittal plane in line with the medial cingulum in both hemispheres (Fig. 4). Mean tensor-based metrics (FA, MD, AD, RD) and free water fraction were obtained.

### Behaviour Assessment

At 20 months of age, mice were assessed for motor deficits in open field, rotarod and spontaneous locomotor activity.

#### The Open field test

The open field test was conducted using Ethovision Pro version 2.3.19 software (Noldus Information Technology, Netherlands) to investigate anxiety-related and exploratory behaviour. Total locomotion, average time spent moving, velocity and rearing frequency was measured in a square test arena (81 × 81 cm) containing 9 equally-spaced quadrants using a camcorder (Sanyo CCD camera) over a 20 minute period.

#### Rotarod

The rotarod test^37^ was used to assess motor coordination, balance and endurance^38^,^39^. Mice were trained and tested on the accelerating rotarod (3.1 cm diameter) apparatus (Ugo Basile Research Apparatus, Varese, Italy). Training consisted of two 5-minute trials on 2 consecutive days; the training protocol involved 15 seconds of fixed speed, followed by a period of accelerating speed, with each successive period increasing in duration. During training, when mice fell off the rod they were placed back on until the full time had elapsed. There were 2 trials on the testing day (the day after the training day), with the speed of revolutions increasing from 4 to 44 r.p.m over a maximum period of 300 s. The two trials were 15 minutes apart. The latency to fall was recorded for each trial.

#### Locomotor activity

To measure spontaneous locomotor activity over the diurnal cycle, mice were transferred to clear Perspex activity chambers (40 cm × 24 cm × 18 cm) containing food and water for 27 hours. The chambers were fitted with 8 infra-red sensors and the total number of beam breaks made in 5-minute intervals was recorded on MED Associate hardware and MED-PC^®^ software (Vermont, USA). Lights were on a 12-hour timer to avoid experimenter interference (light phase [18:00–05:59]; dark phase [06:00–17:59]). Transfer/habituation activity was measured during the first 2 hours; circadian-related activity was assessed over 24 hours (from hour 3 to 27) after allowing for acclimatisation.

### Perfusion

Mice were terminally anaesthetised via intraperitoneal injection of Euthatal (0.1 ml), and then perfused with phosphate buffered saline (PBS, 60 ml) followed by 4% paraformaldehyde (PFA, 150 ml) in PBS (pH 7.3) at a flow rate of 30 ml/min. The skulls underwent post-fixation in 4% PFA in PBS overnight and were transferred to a 25% sucrose solution.

### Histology

After *ex vivo* imaging, brains were removed from the skulls and transferred to a 25% sucrose solution until they sank. Brains were bisected at the midline.

#### Immunohistochemistry

From the left hemisphere, coronal sections of 40 μm were cut on a freezing sledge microtome and stored at −20 °C in tissue cryoprotective solution. All sections were stained at the same time with a 1 in 6 series; sections were first placed in tris buffered saline (pH = 7.4) and washed twice. Solochrome cyanine was used to identify myelin and thus differentiate grey matter from white matter. The tissue was hydrated by serial washes in 100%, 95% and 70% ethanol and distilled water for 5 minutes each and then immersed for 35 minutes in solochrome cyanine solution. Sections were thoroughly washed and differentiation was carried out in 10% Iron Alum for 2–3 minutes, halted by thorough washing. Sections were dehydrated by serial washes in 70%, 95% and 100% ethanol for 2 minutes each, cleared in xylene, and cover-slipped with DPX mounting medium (RA Lamb, Eastbourne, UK).

#### Cortical measurements

Cortical thickness measurements may provide unique information not captured by cortical volume alone^40^,^41^. Changes in cortical thickness may reflect a change in the size, density and arrangement of neurons, dendritic arborisation, myelination change, and neuroglia, therefore cortical thickness can provide unique and important information about the effects of HD not captured by cortical volume alone. Previous work in HD has shown that with increased disease progression, changes were more evident in cortical thickness measures compared to cortical.

Cortical thickness was measured on approximately the same coronal sections for both the MRI images and the prepared brain sections (Fig. 3). Analysis was conducted using Analyze software (version 10.0) for the MRI images and using a Leica microscope at a 5x objective with background correction for uneven illumination for the mounted brain sections. Cortical thickness was measured in primary motor cortex (M1) at approximately Bregma 1.10mm, 0.14 mm, −0.22mm and −1.06 mm.

#### Electron microscopy

200μm thick sections were cut from the right hemisphere on a Leica vibrating-blade microtome (VT1000 S) and stored at 4 °C in 0.1M PBS + azide. The tissue was post-fixed in 2% osmium tetroxide (90 minutes), then dehydrated in isopropanol (50%, 70%, 90%, 2 × 100%, 10 minutes each). Following this, the tissue was infiltrated with embedding resin (50% in isopropyl alcohol for 30 minutes, pure resin 4 × 1 hour, TAAB Laboratories) and then cured for 16 hours at 60 °C. Ultra-thin sections (80 nm) were cut and collected on 300-mesh copper grids, and post-section stained with 4% uranyl acetate and then with Reynolds’ lead citrate. Sections were examined using a Philips CM12 transmission electron microscope (TEM) operated at 80 kV (FEI Ltd, Cambridge) at 4,400x magnification. From each section, 5 images were acquired using a MegaView III digital TEM camera and iTEM software (Soft Imaging System GmbH, Münster, Germany), resulting in a total of 75 images (10 mice [5 Hdh^Q150/Q150^, 5 Hdh^+/+^] × 3 regions × 5 images per region), image area = 327.5 μm^2^. Image analysis was conducted using ImageJ; all axons in all images were counted and the axon density calculated (no. of axons per μm^2^ tissue) with a minimum of 100 axons counted per region, along with the total number of decompacted axons, defined as those with split myelin and/or with distinct, multiple lanullae layers^42^. Sample images and examples of myelin decompaction are shown in Supplementary Figure 4.

### Data Quality Assessments

After visual inspection of the atlas-based segmentation, the globus pallidum and ventricles were removed from the analysis for 2 *Hdh*^+/+^ mice and 2 *Hdh*^*Q150/Q150*^ mice and the thalamus was removed from the analysis for 1 *Hdh*^+/+^ mouse at 7 months due to imperfect segmentation. For *ex vivo* images, the cortex was excluded from the analyses for 5 *Hdh*^+/+^ mice, due to imperfect segmentation and/or damage to the cortex in sample preparation.

### Statistical Analysis

All statistical analysis was performed using SPSS (version 20, IBM, Portsmouth, UK). Extreme outlier values, defined as those over 3 standard deviations from the mean, were excluded case-wise. Due to outlier removal, animal loss and occasional missing values, the number of animals included for each analysis at different time points varied. For post-hoc analyses, data was corrected for multiple comparisons using the false discovery rate (FDR) at 5%.

It was not possible to assess natural attrition across the lifespan as some mice did not recover from anaesthesia. Instead, a chi-square test examined survival during the first 7 months, and from 7–19 months, separately from survival during the two scan sessions.

Weight was included as a covariate for motor function analyses when Pearson’s correlation coefficient between weight and motor performance was greater than 0.3. The assumption of homogeneity of regression slopes was tested; when weight significantly interacted with genotype and the direction of the relationship was the same for *Hdh*^*Q150/Q150*^ and *Hdh*^+/+^ mice, weight was added as a covariate and both the main effects of genotype and the corrected model, encompassing the contribution of weight, are reported.

### Atlas-based segmentation of T2-weighted images

T_2_-weighted images were analysed using an automated multi-atlas based parcellation pipeline^43^. Briefly, the brain was digitally extracted to exclude non-brain tissue by creating a mask from multiple atlas images^44^. The MRM atlas^45^ was selected based on superior parcellation accuracy. The native subject image was globally registered to all atlas images using a block-matching-based symmetric affine registration^46^ and the resulting transformation matrices were inverted and used to propagate the atlas masks to the native image. The resulted brain mask candidates were fused^47^ and this final mask was dilated to include the contrast between brain tissue and CSF. The non-parametric non-uniform intensity normalization technique (N3) was used (see Supplementary Figure 2) and the affinely-aligned atlas images were then non-linearly registered to each mouse image^48^, with the resulting deformation fields used to propagate the labels from atlas space to native space. These labels were fused using the STEPS algorithm^49^. From the labelled images, the caudate putamen, thalamus, globus pallidus, hippocampus, neocortex, cerebellum and ventricles were extracted (Supplementary Figure 2) and the volume calculated. The total *in vivo* brain volume was the summed volume across all ROIs.

## Additional Information

**How to cite this article**: Steventon, J. J. *et al.* Longitudinal *in vivo* MRI in a Huntington’s disease mouse model: Global atrophy in the absence of white matter microstructural damage. *Sci. Rep.* **6**, 32423; doi: 10.1038/srep32423 (2016).

## Supplementary Material

Supplementary Information

## Figures and Tables

**Table 1:** Outcome of t-tests between HdhQ150/Q150 and Hdh^+/+^ mice for absolute volume and regional volumes normalised by total brain volume (TBV).

Region	Volume	7 months			19 months		
		t	df	p	t	df	p
TBV		3.32	42	<*0.005*	4.51	26	<*0.001*
Caudate-Putamen	Absolute	4.08	42	<*0.001*	3.65	26	<*0.005*
	Normalised	1.56	42	>*0.05*	1.33	26	>*0.05*
Cortex	Absolute	3.06	42	<*0.005*	2.84	26	<*0.01*
	Normalised	0.08	42	>*0.05*	−0.5	26	>*0.05*
Cerebellum	Absolute	1.03	42	>*0.05*	0.3	26	>*0.05*
	Normalised	−2.82	42	<*0.01*	−3.29	26	<*0.01*
Thalamus	Absolute	3.48	42	<*0.005*	4.21	25	<*0.001*
	Normalised	1.11	42	>*0.05*	0.46	25	>*0.05*
Globus Pallidum	Absolute	3.09	38	<*0.005*	2.43	26	<*0.05*
	Normalised	1.37	38	>*0.05*	2.04	26	>*0.05*
Hippocampus	Absolute	3.6	42	<*0.005*	4.52	26	<*0.001*
	Normalised	0.6	42	>*0.05*	0.4	26	>*0.05*
Lateral Ventricles	Absolute	2.71	38	<*0.05*	1.42	26	>*0.05*
	Normalised	0.86	38	>*0.05*	−1.19	26	>*0.05*

df: degrees of freedom.

**Table 2:** Axon fibre density and myelin structure in the corpus callosum of the *Hdh* ^*Q150/Q150*^ and *Hdh* ^+/+^ mouse obtained from electron microscopy images.

		Number of fibers measured ± S.D.			Mean axon density (axon/μm^2^) ± S.D.		
		Genu	Body	Splenium	Genu	Body	Splenium
Total	Hdh^Q150/Q150^	1224.4 ± 201.0	1408 ± 443.2	1213.4 ± 414.9	0.75 ± 0.11	0.86 ± 0.26	0.74 ± 0.24
	Hdh^+/+^	1246.0 ± 389.0	932.6 ± 490.1	1199.4 ± 547.0	0.67 ± 0.29	0.57 ± 0.28	0.73 ± 0.32
					% Decompacted (decompacted axons/total axons)		
Myelin decompaction	Hdh^Q150/Q150^	70.4 ± 35.5	146.6 ± 93.3	126.2 ± 108.7	5.96 ± 2.8	11.22 ± 6.8	12.99 ± 12.5
	Hdh^+/+^	60.0 ± 49.3	90.4 ± 66.7	140.6 ± 140.7	7.13 ± 6.3	12.89 ± 9.7	10.62 ± 9.7
