# Supplementary material for: Longitudinal in vivo MRI in a Huntington’s disease mouse model: Global atrophy in the absence of white matter microstructural damage
Source: Sci Rep. 2016 Sep 1;6:32423. doi: 10.1038/srep32423 (PMC5007531; doi:10.1038/srep32423)
Supplement: Supplementary Information [file srep32423-s1.pdf]

Supplementary Information

Longitudinal *in vivo* MRI in a Huntington's disease mouse model: Global atrophy in the absence of white matter microstructural damage

Authors: Jessica J Steventon*[a,b,c, d]; Rebecca C Trueman[b,e]; Emma Yhnell[b,c] ; Zubeyde Bayram-Weston[b,c], Ma Da[g,h,] Marc Modat[g], Jorge Cardoso[g], Sebastian Ourselin[g,]Mark Lythgoe[h], Andrew Stewart[d], Anne E Rosser[b,c,e]; Derek K Jones[a,c]

Affiliations:

a Cardiff University Brain Research Imaging Centre, School of Psychology, Cardiff University, Maindy Road, Cardiff, *CF24 4HQ*, UK.

b Brain Repair Group, Life Science Building, 3rd Floor, School of Biosciences, Cardiff University, Museum Avenue, Cardiff, CF10 3AX, UK.

c Neuroscience and Mental Health Research Institute, Cardiff University, Hadyn Ellis Building, Cathays, Cardiff, CF24 4HQ

d Experimental MRI Centre, School of Biosciences, Cardiff University, Museum Avenue, Cardiff, CF10 3AX, UK.

e School of Life Sciences, Queen's Medical Centre, Nottingham University, Nottingham, NG7 2UH, UK

f Institute of Psychological Medicine and Neurology, School of Medicine, Hadyn Ellis Building, Maindy Road, Cathays, Cardiff CF24 4HQ

g Centre for Medical Imaging Computing, University College London, London, UK ;

h Centre for Advanced Biomedical Imaging, Division of Medicine, University College London, London, UK.

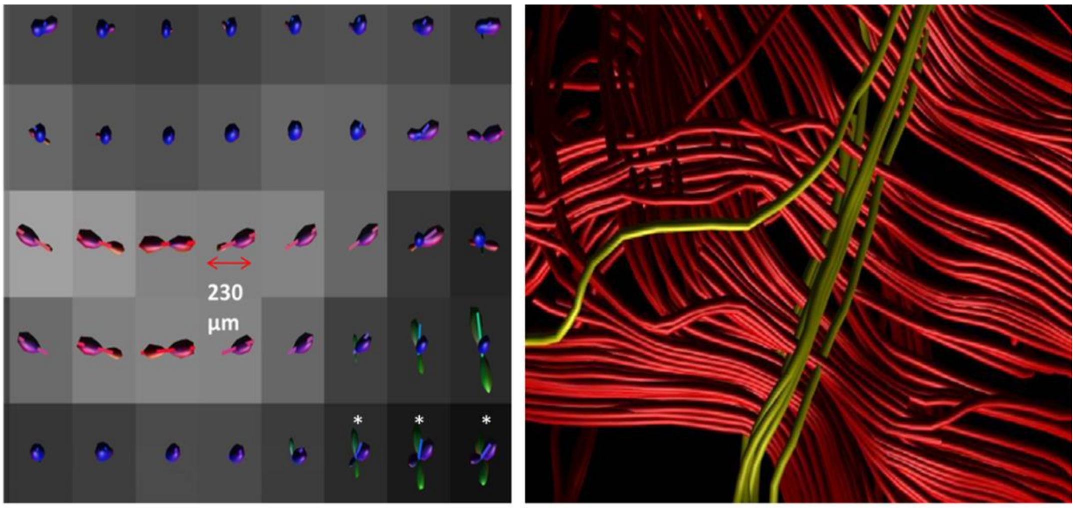

Figure S1. Demonstration of crossing fibers in the mouse brain using spherical harmonic tractography analysis (Tournier et al., 2008). On the left, in each voxel, the principal diffusion direction (sticks) and the fiber orientation distribution (ellipsoids) are shown, the white stars showing examples of voxels containing more than one fiber orientation. Right: Corpus callosum tracts shown in red, cingulum fibers shown in yellow

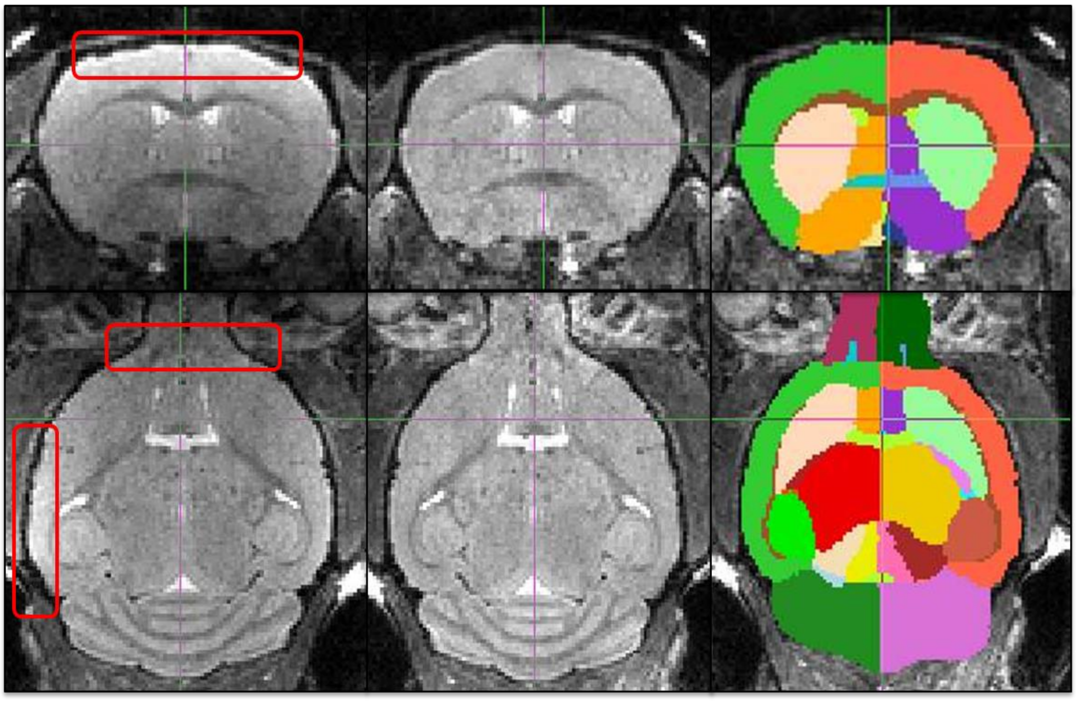

Figure S2 Atlas Based Segmentation of the mouse brain. The left hand panel shows the raw in vivo T_2-weighted image of a representative $Hdh^{+/+}$ mouse brain at 19 months old in the coronal (top) and axial (bottom) plane. The red box shows areas of prominent non-uniformity. The middle plane shows the same brain slice after intensity non-uniformity correction. The right-hand plane shows the labelled regions based on the MRM atlas

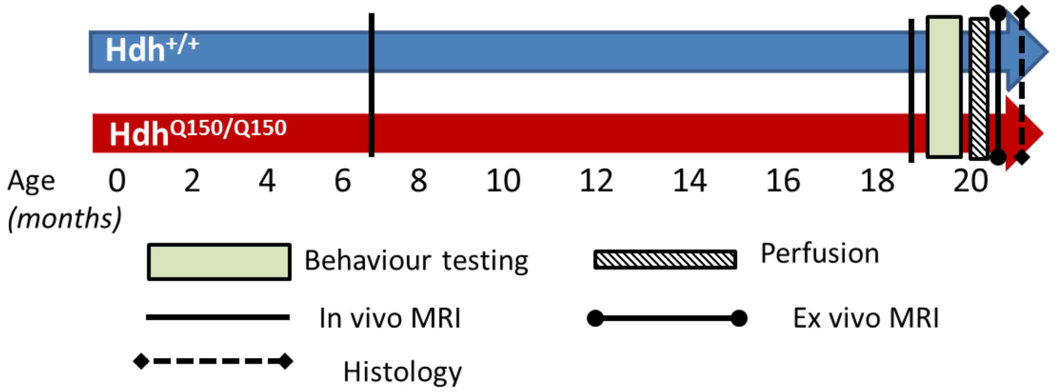

Figure S3. Experimental design from birth to 20 months. Wild-type mice: *Hdh*$^{+/+}$*;* homozygous mice: *Hdh*$^{Q150/Q150.}$

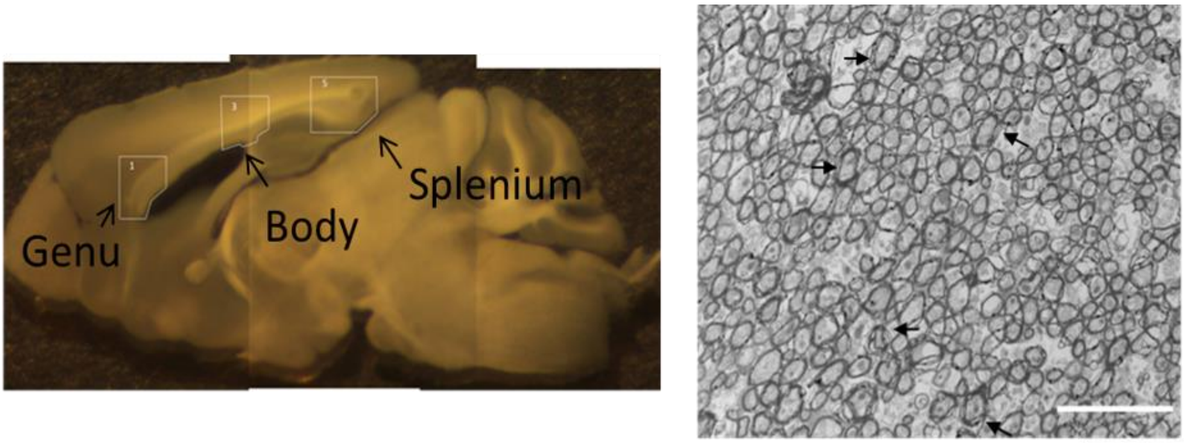

Figure S4. Sample electron microscopy images. Black arrows pointing to examples of myelin decompaction. Scale bar represents 5 µm

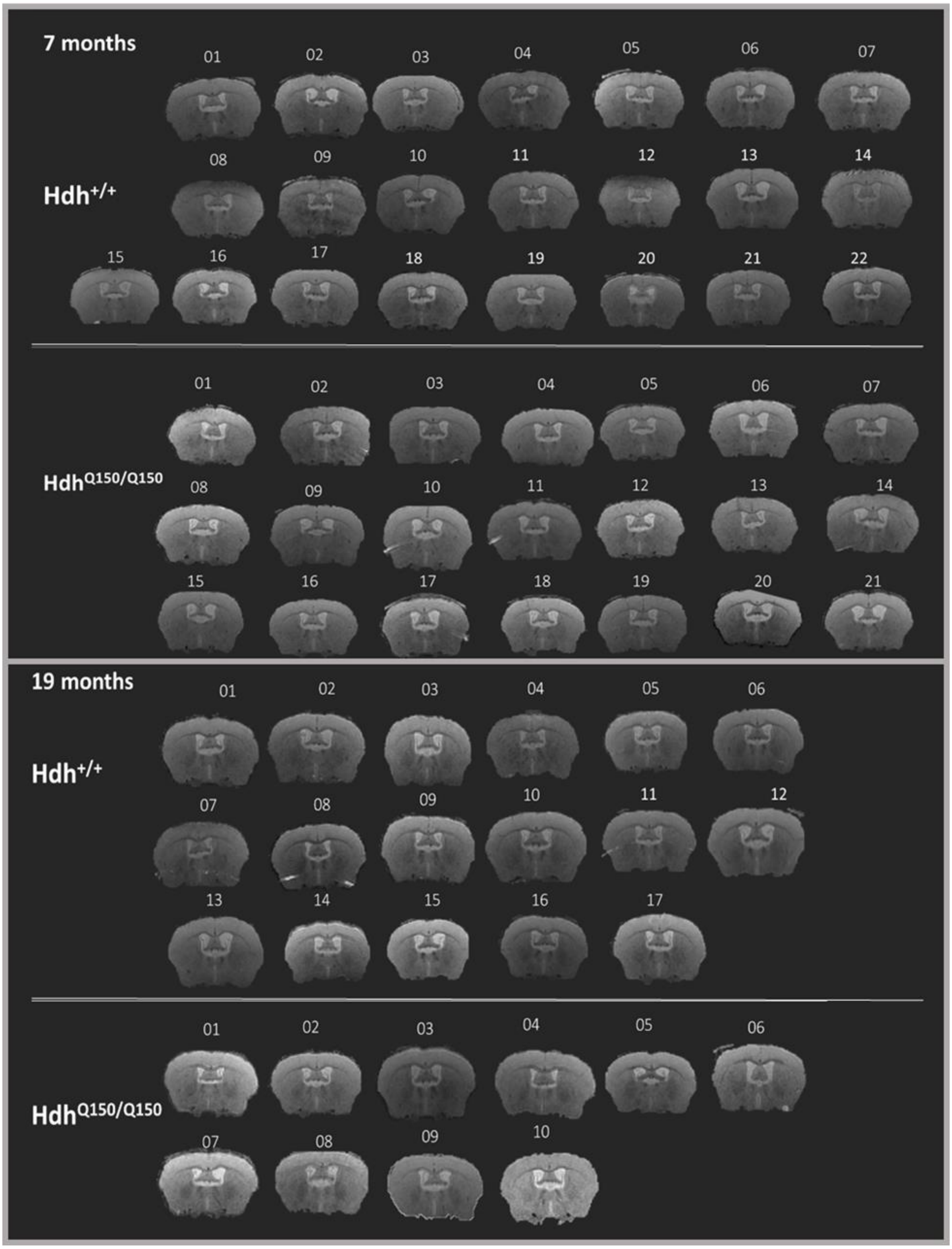

3Figure S5. Raw skull-extracted in vivo T_2-weighted images shown in the coronal plane at approximately Bregma -0.22mm at 7-months of age and 19-months of age.
